# Mapping international research output within ethical, legal, and social implications (ELSI) of assisted reproductive technologies

**DOI:** 10.1007/s10815-023-02834-8

**Published:** 2023-06-29

**Authors:** Ido Alon, Zacharie Chebance, Francesco Alessandro Massucci, Theofano Bounartzi, Vardit Ravitsky

**Affiliations:** 1 Department of Development Economics, Autonomous University of Madrid, Madrid, Spain; 2 University of Montreal, Montreal, Canada; 3 Mines Paristech, Paris, France; 4 SIRIS Lab, Research Division of SIRIS Academic, Barcelona, Spain; 5 Department of Obstetrics and Gynaecology, Faculty of Medicine, School of Health Sciences, University of Thessaly, Larissa, Greece; 6 Harvard Medical School, Boston, MA USA

**Keywords:** Assisted reproductive technologies, Ethical, social, and legal implications, Mapping, Topic modeling, Geographic distribution of research, International research

## Abstract

**Purpose:**

Research about ethical, legal, and social implications (ELSI) of assisted reproductive technologies (ART) is influenced by cultural and value-based perspectives. It impacts regulations, funding, and clinical practice, and shapes the perception of ART in society. We analyze trends in the global literature on ELSI of ART between 1999 and 2019. As most output is produced by North America, Western Europe, and Australia, we focus on international research, i.e., academic articles studying a different country than that of the corresponding author.

**Methods:**

The corpus, extracted from PubMed, Web of Science, and Scopus, includes 7714 articles, of which 1260 involved international research. Analysis is based on titles, abstracts and keywords, classification into ART fields and Topic Modeling, the countries of corresponding author, and countries mentioned in abstracts.

**Results:**

An absolute increase in the number of international studies, and their relative proportion. Trends of decentralization are apparent, yet geographic centralization remains, which reflects an unequal distribution of research funds across countries and may result in findings that do not reflect global diversity of norms and values. Preference for studying conceptual challenges through philosophical analysis, and for fields that concern only a portion of ART cycles. Less attention was dedicated to economic analysis and barriers to access, or to knowledge of and attitudes. International studies provide an opportunity to expand and diversify the scope of ELSI research.

**Conclusion:**

We call on the research community to promote international collaborations, focus on less explored regions, and divert more attention to questions of cost, access, knowledge, and attitudes.

## Introduction

Assisted reproductive technologies (ART) are raising many ethical, legal, and social implications (ELSI) that have been increasingly studied under the lens of humanities and social sciences. In the last two decades, ART practices around the world have increased considerably [1–4]. At the same time, with the global growth in academic publications [5, 6], the volume of research output about both technical and non-technical aspects of ART has expanded substantially [7].

A significant share of research in ART is dedicated to bioethical**,** psychological, sociological, anthropological, legal, and economic perspectives. Assessments of ELSI are influenced on the one hand by cross-cultural differences and value-based perspectives, and on the other, have an impact on the way individuals and societies perceive ART, on the evolution of norms of clinical practices, and on the way these technologies are regulated, provided, and funded [8–13].

As in many other fields, the bulk of global research is mainly produced by a limited number of countries, mostly from North America and Europe, Australia and Japan, and more recently by China and India ([14, 15]; White K., 2019). However, analyzing the geographic distribution of academic research based solely on funding sources and corresponding authors may raise some biased conclusions since it ignores many cases in which the country under research is different than that of the researcher or the funder.

In this paper, we analyze international trends in the global literature concerning ELSI of ART between 1999 and 2019. We focus on the international research output in this field, which we define as those cases in which an academic article has a corresponding author from one country but is studying another. We previously reported on the whole corpus of literature that was extracted from PubMed, Web of Science, and Scopus and included 7714 articles concerning ELSI of ART, i.e., ART applications from humanities and social sciences perspectives (Alon et al., 2023 — under review in *JARG*). Nevertheless, 3741 (48.5%) articles were identified as dealing with a specific country (or countries), of which 1260 (33.7%) were characterized as international research. In the entire corpus, 26% of the articles had a corresponding author from the USA and 14% had a corresponding author from the UK, although the combined leaders’ share decreased from 51% in 1999 to 31% in 2019. Moreover, within international research, 22% had a corresponding author from the USA and 14% had a corresponding author from the UK. More broadly, 81% of the entire corpus had a corresponding author from the leading 15 countries (89% in 1999 and 73% in 2019) (Alon et al., 2023 — under review in *JARG*). Hence, the ELSI literature in ART reviewed in this paper, similarly to ELSI literature in general, is largely shaped by socio-cultural contexts, including economic, religious, and social norms, among other value-based factors, that significantly differ across various societies and circumstances [16, 17]. Since this literature is largely concentrated in a limited number of countries or centers ([7]; Alon et al., 2023 — under review in *JARG*), it may result in findings that are not globally representative or comprehensive. Hence, value-based perceptions from a few countries may shape the framing of and approach towards research questions and analysis worldwide, which may pose a challenge to be addressed.

Geographical concentration of publications stems from unequal distribution of research funds and therefore of researchers around the world. Additionally, some of the world leaders in the practice of ART, such as China, Japan, and Russia, may be producing substantial literature on ELSI of ART in local journals in their languages, literature that large part of it is therefore not being captured by systematic reviews that cover English publications. Their actual contribution to this body of research, and hence the role they could play in the international academic debate, may be underestimated.

Given the unequal distribution of research funds, international research provides a genuine opportunity to expand the geographical scope of research, and diversify cultural perspectives within the global discussion surrounding ART, which is particularly relevant for the value-based ELSI disciplines. International research may be perceived as potentially imposing the views of researchers from well-funded countries regarding, for example, what value ought to underline the field or what research questions are worth exploring. This could promote patronizing attitudes towards countries where research funds are limited, and cultural values differ from those in rich Western countries that dominate the field. However, international research can also encourage foreign researchers to familiarize themselves with local settings and become better embedded in local cultural contexts. It could also promote a richer research agenda by challenging local social values, asking novel questions from an external perspective, and offer diverse views and insights. Furthermore, what we describe here as international research often takes the form of research collaboration that involves co-authorship among researchers and institutions from different countries, at times including local researchers. Such international collaborations have become more common over the past decade, reflecting an expanding recognition of the significance of diverse perspectives in research [6].

Our aim in this paper is to analyze research links between countries, by crossing the country/es of corresponding author/s with the country/es mentioned in the abstract, as an indicator of the research subject. Additionally, we analyze co-authorships involving different countries. To enrich the analysis, we identify shifts in research focus by dividing the corpus into ART fields and by using Topic Modeling (TM) with Latent Dirichlet Allocation (LDA). Our findings show gaps and opportunities for international research concerning ELSI of ART.

## Methods^1^

### Design

We designed inclusion criteria to select articles dealing with ELSI of ART and exclusion criteria to exclude articles dealing entirely with clinical and medical matters, as described in Appendix A.

### Collection

The corpus was collected from the online databases PubMed, Web of Science (WoS), and Scopus. Since we aimed to analyze titles, abstracts, and keywords, we included article that had an abstract in English, regardless of the article language.

Following a keywords frequency analysis, three groups of Medical Subject Headings (MeSH)-terms were selected, as shown in Appendix B. Group A included ART terms and Group B included terms indicating a relation to ELSI across disciplines within humanities and social sciences. Group C was formed to exclude irrelevant articles. We aimed to find balance between false-positive (inclusion of articles with medical-clinical nature) and false-negative (exclusion of articles concerning social sciences and humanities). We used the PubMed API [18] to query for articles with “One MeSH-term from group A” AND “One MeSH-term from group B” AND “Humans (MeSH)” AND “1999-2019” NOT “Any MeSH-term from group C.”

The PubMed query brought up 11,246 results of which 7003 had a full record of title and abstract in English. Additionally, 159 articles which were queried with no full record from PubMed were imported from Scopus. In total, 7162 articles had a full record.

We dropped all abstracts with less than 50 words (259); removed articles if article type included “Clinical Trial,” “Controlled Clinical Trial,” “Randomized Controlled Trial,” or “Validation Study” (536); and excluded all journal related to biodiversity (34). 6333 articles remained, we extracted a list of keywords including their frequencies within titles and abstracts and divided them to three groups of keywords (see Appendix B) with similar definition as described above. We queried the WoS and Scopus APIs for articles of which the title, abstract, or keywords had “One term from group A” AND “One term from group B” AND “1999-2019” NOT “Any term from group C.”

We extracted 14,394 and 12,588 articles from WoS and Scopus, respectively. In addition to the 6333 extracted from PubMed, 33,315 articles were merged from all three databases. Following the removal of duplicates of titles, abstract, and DOI, 17,247 articles remained, of which 14,283 had available title and abstract in English. We repeated the cleaning methods previously applied on the PubMed query (explained above), and removed 154 articles due to short abstract (less than 50 words), and 99 from journals of biodiversity. 14,030 articles remained.

### Manual Cleaning

Two researchers cleaned up the corpus by analyzing the titles and abstracts with an emphasis on the rejection criteria from Appendix A. We removed 6315 articles and remained with a database of 7714 relevant articles of full record. 1184 were non-English articles, with English abstracts.

### Classification

The abstracts were processed by merging the title and abstract into one string (“code”), harmonized, tokenized^2^, and lemmatized. We formed a list of terms to be replaced with acronyms or abbreviations in order to unify the text and allowing us to identify repetitions. Next, we extracted a list of terms by the frequency of “codes” in which they appear and divided the most frequent technical-medical terms into 10 ART fields (see Appendix C): (1) Egg Donation; (2) Sperm Donation; (3) Embryo Donation; (4) Surrogacy; (5) Fertility Preservation; (6) Stem-cell Research; (7) PGT; (8) Genome Editing; (9) MRT; (10) Assisted Insemination. An article was assigned into an ART field (or several) if one term, associated to that field, was found in its title, abstract, or keywords. The articles which included none of these terms were assigned by two researchers reading their titles and abstracts. The remaining articles (2267/7714) were appointed as “General.”

Subsequently, for each publication, all obtainable metadata was extracted from WoS, Scopus, and PubMed, merged and unified under one template. Finally, we cleaned the abstracts from all those technical-medical terms that were used to define the 10 ART fields and then: (1) Uploaded the database to the VOSviewer software tool for constructing and visualizing bibliometric networks. (2) Applied Topic Modeling (TM) via Latent Dirichlet Allocation (LDA) (Blei, Ng, and Jordan 2003; Kapadia, 2019) to all “codes.” To do so, we have built a corpus dictionary using gensim^3^ open-source library.^4^ The LDA method assumes that the observed distribution of words in a textual corpus is determined by a statistical model that fixes both a word-topic and an article-topic association [7].

**Fig. 1:**
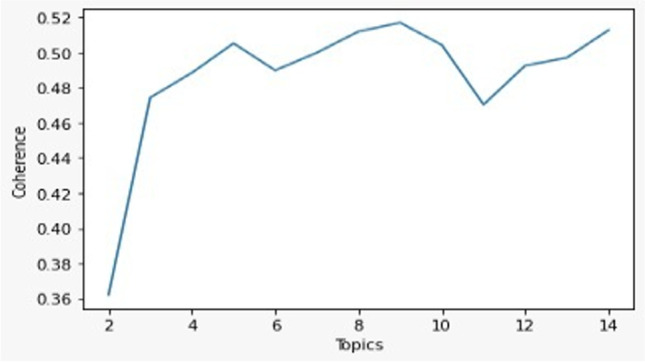
Coherence score as a function of the number of topics

We defined the number of topics by computing the coherence score as a function of the number of topics^5^ and by assessing the results in relation to the VOSviewer analysis. The results of the LDA algorithm consist in a list of topics, and in the weighted relations (0 to 1) between each article and each topic. Every topic is a list of characterizing words (reported in Appendix E), and each article may be connected to more than one single topic. Thus, as seen in Fig. 1, at least 5 topics should be identified. According to our analysis, we defined 6 topics (Fig. 4) as the most accurate solution: (1) Bioethics, (2) Cost and Outcome, (3) Law and Policy, (4) Psychology, (5) Family and Sociology, (6) Attitudes and Knowledge. Each article was associated with those topics by considering a weighted relation of more than 0.333334, so that each article could be associated with not more than two topics. As a result, only 161 articles remained unassociated.

### Analysis

In addition to the VOSViewer tool, we conducted an analysis based on the year of publication, countries of corresponding^6^ authors, and countries mentioned in codes as an indicator for the focus of the article. For the last, we searched the codes (abstracts, titles, and keywords) using a list of countries, cities (with no duplications), and nationalities.^7^ Furthermore, we analyzed the database according to the predefined ART fields, the topics identified by the TM, and regions^8^.

We tested for Spearman’s correlations between the ART fields and the topics (Appendix 4). Those correlations were used for two purposes: first, to group ART fields in order to simplify the presentation of results, i.e., [Egg (C1) and Sperm donation (C2)], and [Stem-cell Research (C6) PGT (C7) and Genome Editing (C9)]; second, to verify a relationship between ART fields and topics.

## Results

**Fig. 2:**
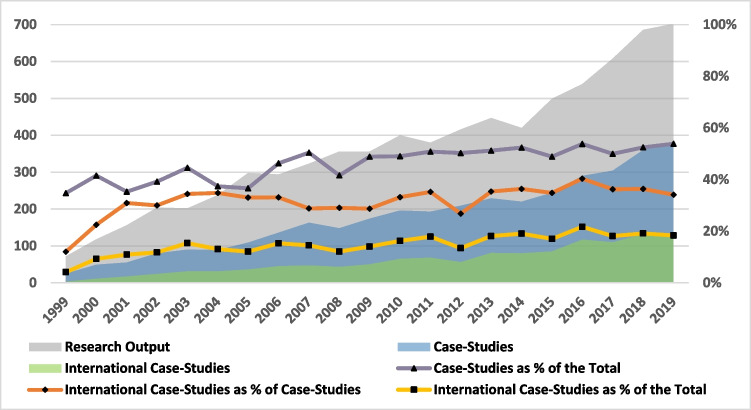
Trendline of output and international research

The total global research output in ELSI of ART grew from 72 publications in 1999 to 702 in 2019 (the gray area in Fig. 2). The share of publications that were dealing with a certain country (or several), which we label as “case-study,” was 39% in 1999–2005 and increased to 52% in 2013–2019 (the purple trendline). Among those “case-studies,” the share of “international case-studies” increased from 31% in 1999–2005 to 36% in 2013–2019 (the orange trendline). Therefore, the increase in the “international research” as a share of the total literature (the bottom yellow trendline) is mainly due to the increase in the share of “case-studies.”

### Areas of international research

**Fig. 3:**
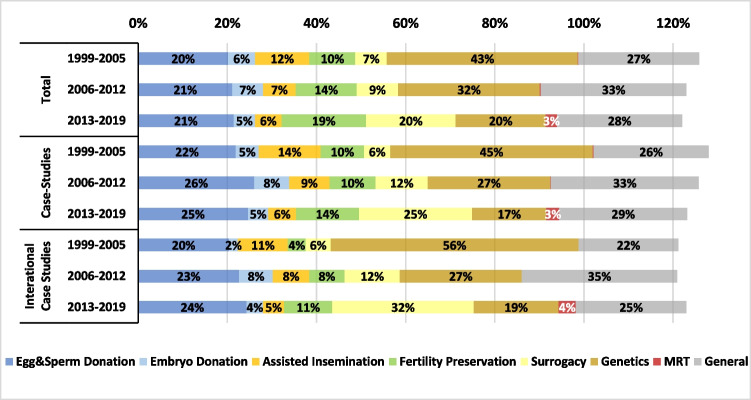
Research by ART categories

**Fig. 4:**
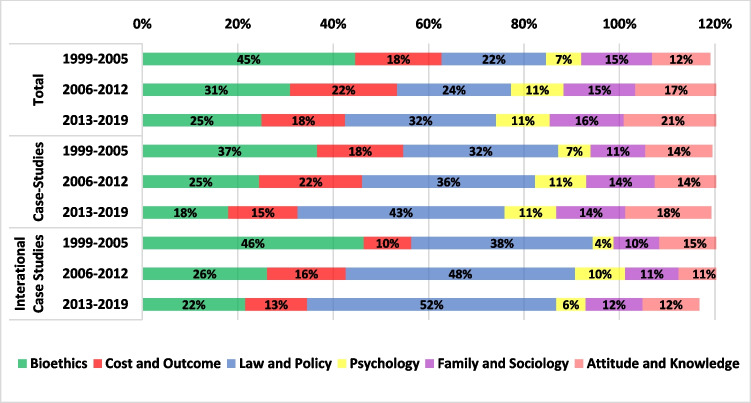
Research by topics

Figures 3 and 4 present the distributions of 10 ART fields and 6 topics, respectively, allowing to observe the differences in trends between the entire literature, case studies in general, and international research. Some articles were assigned to more than a single ART field or a single topic. For this reason, the total sums up to more than 100%.

The field of “Egg & Sperm Donation” was increasingly elaborated under case studies in general. The field “Fertility Preservation” was researched in general terms more frequently than under a certain geographical context. Nevertheless, in the last 7 years, this field was more often engaged by international research. Along the timeline, “Surrogacy” became the central subject of international research while “Genetics,” which was highly engaged at the beginning of the period, moved out of the spotlight. These trends were evident for the entire corpus, but were particularly noticeable in international research.

As seen in Fig. 4, the topic “Bioethics” had decreased prominence under case studies and under international research compared with the general literature. In contrast, “Law and Policy,” which was the growing and most central topic at the end of the period for the entire literature, was more intensely engaged by international research. Hence, a comparison of laws and regulations between countries has become one focus of the literature over time. International research of the other topics was more limited, mainly concerning “Psychology.” Nevertheless, due to the inclusion criteria for the database selection, this topic includes only articles with ELSI or impact on patients’ decision-making and not all psychological research concerning ART.

### Producers of international research

**Fig. 5:**
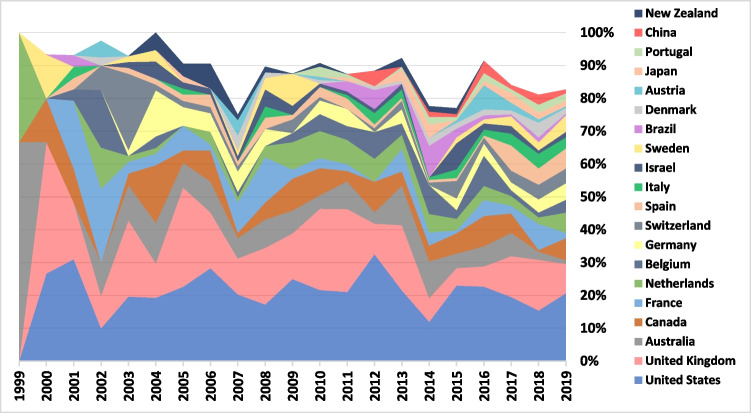
20 top producers of international research

Figure 5 shows the top 20 producers of international research. In the first few years under study, 5–15 countries were responsible for nearly all international research production. However, at the end of the period, the top 20 countries (Fig. 5) were responsible for the production of 83% of international case studies, while a few more emergent producers (Russia, Ukraine, Taiwan, Greece, and Romania) presented several publications (more than 5 each, in 2019). For the two leaders, the USA was ahead in publications throughout most of the period and kept stable while the UK lost a significant share.

### Subjects of international research

**Fig. 6:**
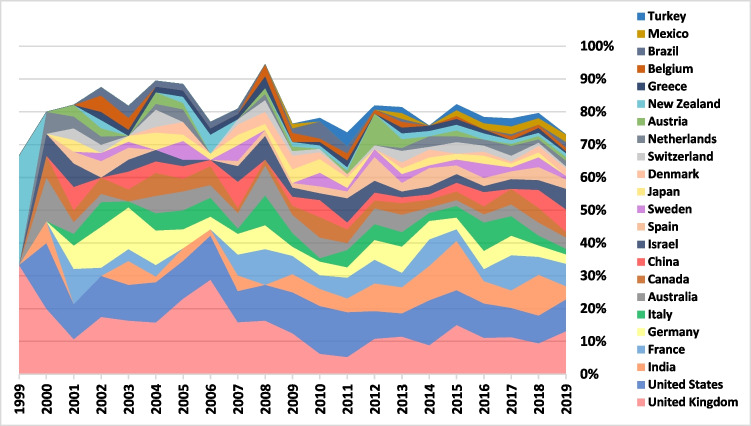
23 top subjects of international research

Figure 6 shows the top 23 countries that were international research subjects (were mentioned in at least 20 publications throughout the period). The UK was regularly the most popular subject for international research. Among some of the emergent research subjects, we notice India, which became the third most studied, China, the world leader of ART, Israel, Turkey, and Mexico.

### Pivot analysis

Tables 1, 2, and 3 in Appendix E combine a pivot analysis of ART fields and topics in international research, allowing to identify some major trends and reveal which countries are conducting research about which countries, and in which ART fields and topics.

**Table 1:**
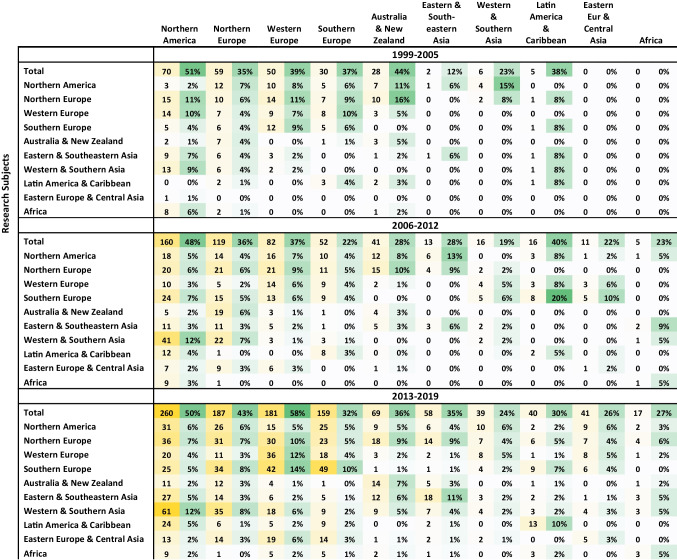
Matrix of regional international research

The matrix shows the total number of international publications produced by each region with their share out of the total case studies produced by that region

**Table 2:**
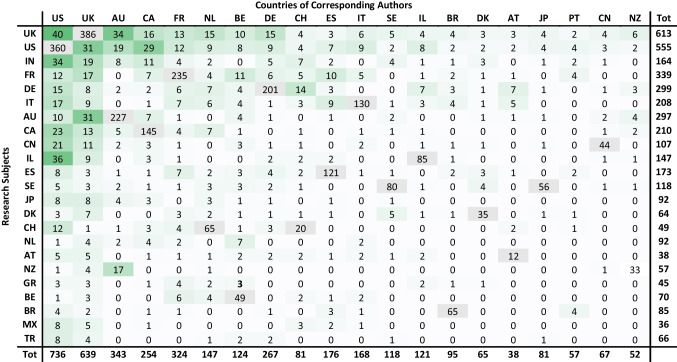
Matrix of international research

**Table 3:**
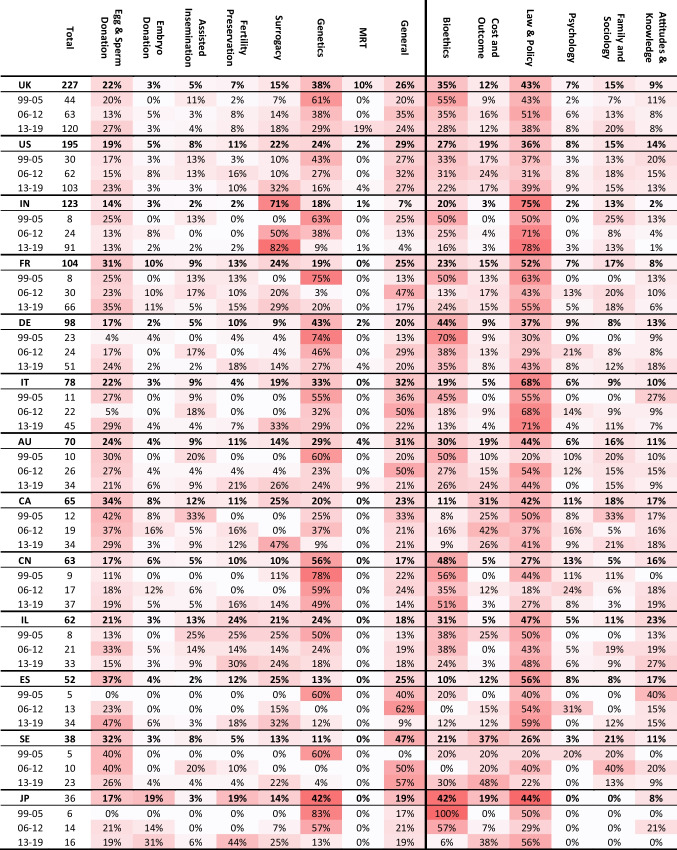

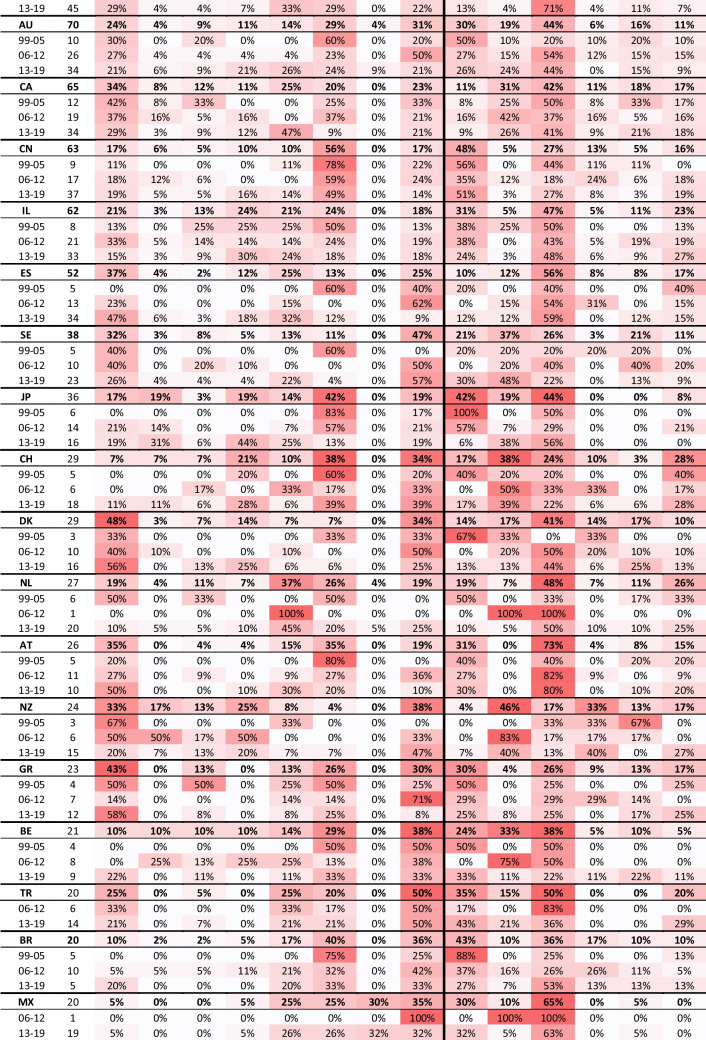
Top 23 subjects of international research by ART fields and topics

Table 1 in Appendix E presents the number of international publications produced by each region in relation to the region under study. It also presents the percentage of international publications as a share of all case studies produced by that region. The Northern American Region, which is constituted of only two countries, is the leader of international publications. A large share of it studied Western and Southern Asia, particularly India and Israel, but Northern American researchers were also engaged in research about Europe, Eastern and Southern Asia, and Latin America.

The Northern and Western European Regions have increased their production of international research while the Southern European share declined over time. Moreover, Western European nations were increasingly producing research within their continent while Southern European nations produced their research even closer to home, mainly within their own region. In contrast, international research by Northern Europeans was gradually more diversified in geographical terms. Additionally, Latin American research increased over time and a large share of it was exchanged within the continent. Eastern European and African countries had increasing output and growing participation in international research.

On a national level (Tables 2 and 3 in Appendix E), the exchange between the two leaders was the strongest among all nations. The US research about the UK was strongly centered on the field “Genetics” throughout most of the period, with a focus on the topics “Bioethics” and “Law and Policy.” In the opposite direction, the UK research on the USA was more diversified, with a light focus on similar topics and the addition of “Family and Sociology.” The UK and the USA, as research subjects, attracted many researchers from Australia, Canada, and European countries. In the first 7 years, such international research in the UK was extremely focused on “Genetics,” “Bioethics,” and “Law and Policy,” but later the field “Surrogacy” gained share. The research about the USA was more diversified.

India was the next popular research subject, as 75% of the research mentioning it was done by a foreign corresponding author. It mainly addressed the topic “Law and Policy” and was strongly approached by researchers from the USA (21%) who were intensely focused on “Surrogacy.” In total, 112 articles addressed surrogacy in India, which is 12.6% of all articles dealing with (ELSI of) surrogacy, and 68% of those dealing with India.

In South Europe, studies about France and Italy have been quite diverse with a growing interest in “Surrogacy” and a modest focus on “Egg & Sperm Donation.” Research about Spain has become much more focused on these two ART fields with an emphasis on the latter. Moreover, the research on this region was strongly engaged with “Law and Policy.” Except for the engagement by the two leaders, USA and UK, a large part of the international research in South Europe was actually exchanged within the region, although France was also often researched by Belgium under the field “Egg & Sperm Donation.”

Research about Canada had a diminishing interest in “Egg & Sperm Donation” and an emerging interest in Surrogacy over the last 7 years, with a focus on the topics “Law and Policy” and “Cost and Outcome.” Germany was intensely researched by Switzerland (about “Fertility Preservation”) and Austria, while Australia was engaged mainly by researchers from the UK.

Research about China and Brazil was extremely focused on “Genetics” and “Bioethics.” It became slightly more diversified with time, although more for Brazil than for China, where it remained the focus of 49% of international research in the final 7 years. Japan, the second largest operator of ART, was also a popular research subject under the field “Genetics” and the topic “Bioethics” between 1999 and 2012. However, in 2013–2019, the focus shifted towards “Fertility Preservation” under the topics “Law and Policy” and “Cost and Outcome.” China, Japan, and Brazil were mainly approached by researchers from the USA and UK.

In Western and Southern Asia, Israel was the second most popular subject following India, under a diverse portfolio of ART fields and topics, as it was mainly approached by researchers from the USA. Together, India and Israel were the target of 75% of this region’s international research, as a subject. The most common research subjects in Latin America were Brazil and Mexico, which were studied by researchers from the USA and the UK, with a focus on MRT. Costa Rica and Chile were also studied by researchers from the Latin American region. In Eastern Europe, Turkey, the Czech Republic, Poland, Russia, and Ukraine were the subject of a few studies, with a focus on Surrogacy. Lastly, in the last few years, South Africa, Egypt, and Kenya, as subjects, attracted some international researchers to Africa, mainly from the USA.

### Co-authorship

**Fig. 7:**
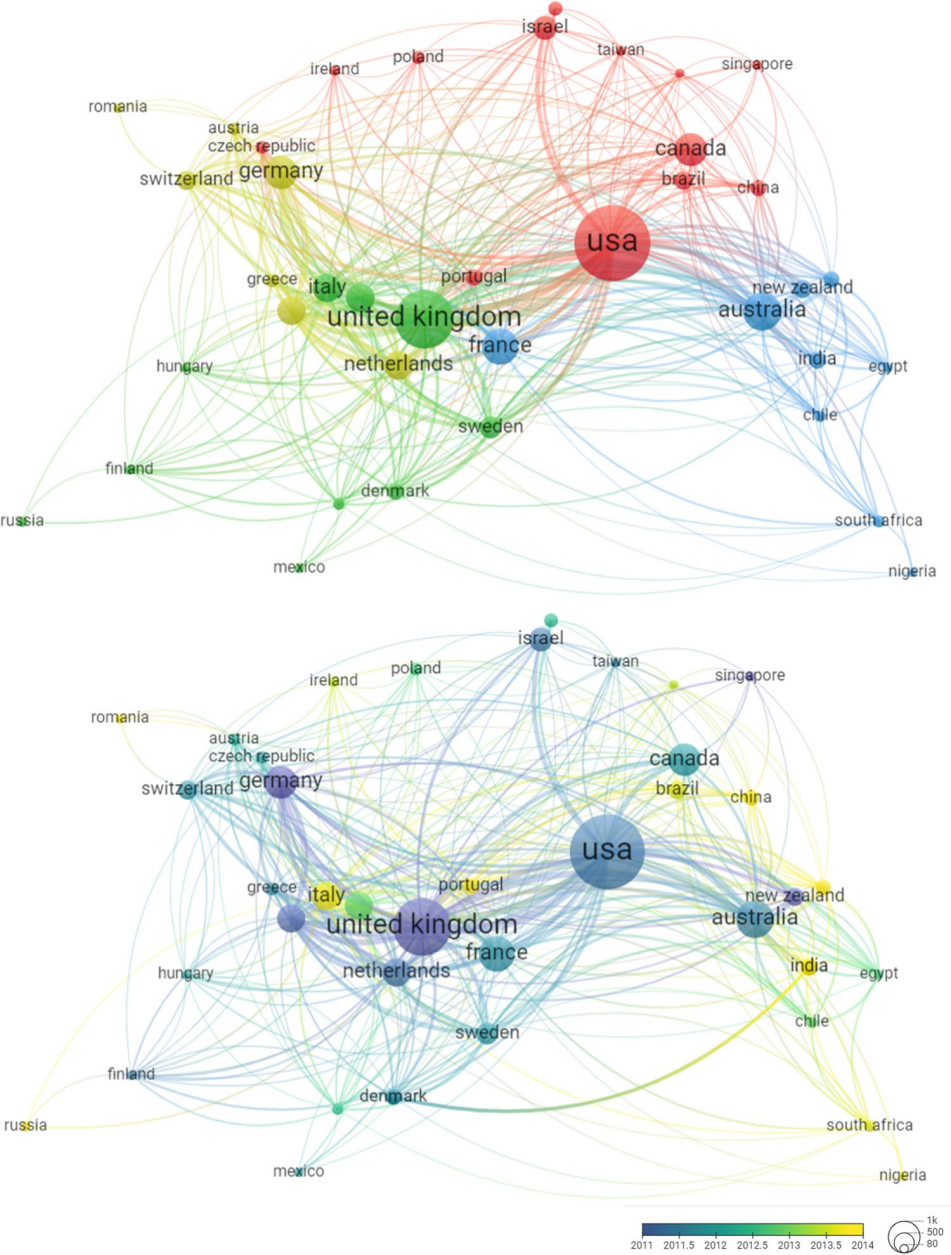
Co-authorship between countries

In addition, we used the VOSviewer tool to analyze co-authorships between countries.^9^ We invite the reader to consult the interactive version of this figure, available online at: https://app.vosviewer.com/?json=https://drive.google.com/uc?id=1YWviVOhhsOSh7VkeTj2JaPrZyyNgDZnR;

In Fig. 7, we may identify four clusters: one led by Northern America which has strong research links with Brazil, China, and Israel; a second cluster led by the UK which is firmly linked with Northern and Southern Europe; third, a cluster for Western Europe, and finally, a cluster led by Australia which is linked with India, Japan, and Africa. Contrary to our previous analysis, the co-authorship between countries does not explicitly reveal anything about the countries being research subjects, but only might imply that such collaborations enable and even stimulate international research. It is therefore not an unambiguous indicator for international research. Nevertheless, it allows an interesting glimpse into international collaborations in ART research.

## Discussion

Between 1999 and 2019, research output on ELSI of ART increased nearly tenfold (Fig. 2), indicating a much higher growth rate compared with clinical research in ART and with the global scientific output [5–7]. In this paper, we explored international research by considering the country of the corresponding author and the country mentioned in the abstract. We noted an absolute increase in the number of international studies, as well as an increase in their relative portion of the whole literature, which rose from 12% in 1999–2005 to 19% in 2013–2019. It is largely explained by an increase in the share of articles representing case studies, i.e., discussing specific countries. Thus, 33% of all case studies in the corpus and 36% of the case studies in the last 7 years (2013–2019) involved a corresponding author from one country studying a different one, which shows that international research about the ELSI of ART is widespread. Nevertheless, as we have shown, large part of this research was exchanged between the core countries and within the leading regions.

According to the US National Center for Science and Engineering Statistics, international co-authorships in global sciences increased from 17 to 23% between 2008 and 2018 [6]. An analysis of co-publications showed a continuous increase in international collaborations between 2000 and 2015. It concluded that the globalization of science is an on-going process that is changing the power dynamics underlying the scientific enterprise and reshaping the global scientific landscape. More countries are participating, ties between countries are becoming more frequent, and international collaboration once led by Europe and the USA is gradually replaced by a tri-polar world comprising Europe, North America, and the Asia-Pacific region [15].

A previous analysis of ELSI research in personalized genomic medicine from 2008 to 2012 revealed that, of 135 ELSI articles in English, 90 (66.7%) focused on ELSI issues outside the USA [19]. Only a limited number of articles discussed Asian and African countries. In contrast, the ELSI of ART corpus from 1999 to 2019, collected in this paper, represents a more decentralized literature. A total of 85.2% of the articles examined ELSI issues outside the USA, with about 25% discussing Asian countries and less than 3% discussing African countries. It is important to note that 15.4% of the corpus is non-English. More countries were producing international research and positive trends of decentralization are apparent, in relation to the countries of corresponding author and the countries under study (Figs. 5 and 7) as well as the countries that are the subject of such research (Fig. 6). It is too early to state that the European-American hegemony has been dismantled. Despite the relatively high share of international research and the rise of India as a research subject, as well as a growing interest in China, we saw that a large share of international studies involved the leaders (USA and UK) and Northern, Western, and Southern European countries.

In Europe, we may identify a link between research funding and international research (Table 1 in Appendix E). The richest region, Northern Europe, was more geographically diversified in its research, and Western European research was conducted mainly within the continent, while the Southern European nations with the least academic resources conducted more international research within their own region. This allows interesting insight into the relationship between funding and research topics. The more resources one has, the more one can “afford” to study regions and countries that are different than one’s own. Hence, increasing research funding and capacity may help diversify research topics and fields globally and allow research attention to be dedicated to areas that are currently understudied.

### Research collaborations

We identify a few international trends in the literature that are compatible with global trends [15]. To some extent, geographic distance is important as neighboring countries often collaborate with each other. However, peripheral countries, in terms of scientific output, are more likely to collaborate with the core nations instead of favoring partnerships with other nearby peripheral countries. Thus, on the one hand, we notice some pairs of frequently collaborating neighbors such as the USA and Canada, France and Belgium, France and Spain, Germany and Switzerland, Germany and Austria, Australia and New Zealand, and Denmark and Sweden.

On the other hand, “peripheral countries” such as India, China, Japan, Brazil, Turkey, and Mexico were mainly studied by and collaborated with researchers from the core nations (see Fig. 7 and Table 2 in Appendix E). Hence, there were strong and growing research links between North America (mainly the USA), and North Europe as research producers, and Western-Southern Asia (mainly India and Israel), as research subjects. This was also seen between Western and Northern Europe as research producers and Easter Europe as a research subject.

In the last 7 years of study, 2013–2019, there was also an increase in the production of international research within Eastern and Southeastern Asia and within Latin America (Table 1 in Appendix E). In those years, in 18 cases, researchers from Japan, Taiwan, China, and Singapore conducted international research about China, Vietnam, Japan, Thailand, Singapore, and South Korea. In Latin America, 13 international articles had corresponding authors mainly from Brazil and were focused on Costa Rica, Chile, and Argentina.

This landscape suggests that it remains important to encourage researchers to explore understudied regions and address topics that are highly dependent on cultural contexts. Ethical, social, and legal issues are often framed and addressed in ways that are deeply embedded within local cultural assumptions. International research that explores the ELSI of ART in diverse cultural contexts therefore has particular potential to shed light on the reasons for differences and discrepancies across the world. It can also point to ways to overcome such discrepancies in areas where harmonization and standardization of policy and of clinical norms can enhance the protection which are vulnerable parties involved in ART (such as children conceived via ART, surrogates, and egg donors).

### Trends in international research areas

Several trends emerged in relation to the fields explored by international research. First, “Egg & Sperm Donation” was highly and increasingly studied through case studies, i.e., in a national context, both concerning research that was produced by authors from the country under study or by another (Fig. 3). The share of international research dealing with this field increased over time, for China and Japan and all European countries (which appeared in Table 2 in Appendix E), except for Sweden and the Netherlands. It was particularly high for Spain and Denmark.

Considering the aging of populations and delayed childbearing in Europe and East Asia, as well as the global crisis in terms of male infertility [20], it is easily understandable why egg, sperm, and embryo donations are a popular subject for a case studies. A very large share of ART cycles, particularly in Europe, involve third-party reproduction, with Spain dominating the market for donor eggs and Denmark that of donor sperm. At the same time, regulations and clinical norms in this context vary substantially, for example, the rising interest in Italy was possibly affected by the 2014 rule of the Italian Constitutional Court which overturned the ban on gamete donation [21]. Moreover, these practices raise multiple socio-ethical tensions involving all stakeholders: ART users, donors, children conceived, and society at large. These tensions often touch on key issues such as the definition of parenthood, parental right and responsibilities, or the rights of children to know their genetic origins. It is hence clear why these issues attract the attention of ELSI scholars.

Second, the engagement of international research with “Surrogacy” has grown remarkably throughout the study period. In the last 7 years, a third of all international research output explored this field (Fig. 3). Specifically, 31% of international publications on surrogacy focused on India, with 5% on Thailand. This increased interest in surrogacy could be partially attributed to the ethical tensions and inequalities based on class, race, and nationality, surrounding the use of surrogates from low-income countries by couples from high-income countries [22], especially when access to surrogacy is limited in the commissioning couple’s country of origins. Furthermore, international research on surrogacy also concentrated on the USA, France, Netherlands, Canada, Spain, Italy, and Austria. The bans on surrogacy in the last three countries may have prompted discussions on this matter, potentially explaining the research focus on these nations.

Surrogacy indeed raises inherent and deep socio-ethical and legal issues, such as the definition of motherhood or the potential exploitation of vulnerable women. It thus deserves the attention of ELSI scholarship. As Pande notes: “A global and complex issue like surrogacy cannot be resolved or regulated within national borders. A global issue like surrogacy needs a global dialogue” [23]. At the same time, since surrogacy represents an extremely low share of ART cycles, this level of engagement may seem rather disproportional, especially in light of gaps in research on issues that are inherent to every aspect of ART, such as cost and access. While we are fully aware of the importance of addressing local issues that are ethically fraught and threaten human rights, considering limited research resources, we cautiously call attention to this focus on a practice that is overall clinically marginal, at the expense of others that have implications for millions of ART users, such as research on attitudes towards and knowledge about infertility and ART.

Third, we demonstrated a notable decrease in the proportion of research addressing the field of “Genetics,” particularly prominent between 1999 and 2005 for the entire corpus, and even more so across international research (Fig. 3). From 2006 onward, the focus on “Genetics” gradually diminished, becoming more domestically focused with fewer case studies and less international research. An exception lies in the growing study of ELSI surrounding MRT, particularly regarding the UK, as it was the first country to regulate this practice [24]. Mexico has also attracted attention due to the 2016 controversy surrounding the birth of the world first MRT-conceived baby [25].

The declining focus on “Genetics” is a surprising, considering the technological advancements during this period, including improvements in stem cell and regenerative medicine, the increased addition of PGT (mainly PGT-A) to ART [1], and the developments in germline genome editing using CRISPR [26]. Several hypotheses can be proposed for this decline. The period between 1999 and 2005 was influenced by various factors generating heightened interest in genetics, such as the cloning of the first mammal — Dolly the sheep — in 1998, the establishment of the ELSI program at the NIH [27], the completion of the human genome project in 2003, and the growing acceptance of PGT as a supplementary technique for ART. The decline after 2005 could be attributed to the normalization of certain technologies, the establishment of regulatory frameworks, and the integration of ELSI perspectives into broader discussions, consequently leading to a shift in research focus.

Another potential explanation is that ELSI researchers have become more cognizant of common concerns already addressed by the literature, such as consent, risk-benefit analysis, informed decision-making, and data sharing. As a result, they may have avoided duplicating publications where existing ethical principles and guidelines were readily applied to new technologies. Furthermore, the increasing domestic focus could be linked to an emphasis on local policies, cultural frameworks, and socio-economic challenges reflecting the needs that emerge when technologies are being implemented into the clinical context.

Indeed, this decline in “Genetics” is only relative, and in absolute terms, the number of articles did increase over the last two decades. Still, we would have expected a steeper growth even compared with other fields. In this context, it is important to mention that in such areas of emerging biotechnologies, some key publications are not academic peer-reviewed papers captured by our analysis, but rather gray literature including influential reports written by leading groups, institutions, or associations [28–30] due to urgency of needs for policy guidance.

In terms of topics, the decrease in “Bioethics” could be explained by the decrease in the field “Genetics,” as the two are correlated — much of the funding of bioethics came from the ELSI program of the NIH [27] and similar funding programs in Europe, which all focus on genetics. Additionally, we examined the effect of excluding articles co-tagged with “Genetics” and discovered that the decrease in “Bioethics” is even more strongly associated with “Genetics” in the context of case studies and international research. Consequently, we propose that there is potential for further research in the field of “Genetics” under different topics, especially “Cost and Outcome,” and “Attitudes and Knowledge” in relation to service accessibility, cost-effectiveness, financial burden, and public education. For instance, in the case of PGT, it would be worthwhile to investigate, under varying healthcare systems, the number of families and individuals who are unable to access this technology, and to examine the implications of this limited access for health outcomes, equity, and societal well-being.

Our analysis shows first a strong increase in the engagement of international research with the topics “Cost and Outcome” from 10% in 1999–2005 to 16% in 2006–2012 and then a decline to 13% in 2013–2019. As seen in Fig. 4, during the period under study, the topic “Cost and Outcome” was engaged more domestically than through international research. Concerning “Attitudes and Knowledge,” there is a noticeable contrast between its rising share in the entire corpus, which increased from 12% in 1999–2005 to 21% in 201–2019, and its decreasing share in international studies, where it dropped from 15 to 12% (Fig. 4). These topics are of paramount importance, and while the overall increase in interest in “Attitudes and knowledge” is encouraging, the decline in international research is problematic. This decline may generate gaps that can negatively impact ART users, clinicians, and policy makers. Addressing “Attitudes and Knowledge,” a topic that represents research on people’s knowledge of and attitudes towards infertility and ART, is critically important under an international context. This includes comparative analysis, as infertility rate upsurge worldwide due to factors such as reproductive age, life style, and pollutants [31, 32]. ELSI research ought to address the global and international aspects of these issues, in particular public education about the causes, implications, and social impact of infertility and the use of ART. Such issues are embedded in cultural values and attitudes, as well as social norms, and thus necessitate the methodologies and conceptual analysis that ELSI of ART research can bring to the field.

Concerning “Cost and Outcome,” it is important to note that this topic may be less explored by international research due to the inherently local nature of costs and the significant variations in policies regarding public funding of ART across countries. Additionally, the overall economic circumstances of each country impact both the availability of public funding and the ability to pay for ART out of pocket. As a result, while international comparisons can be interesting, their value might be limited unless local circumstances are carefully considered and controlled for. Nonetheless, in many countries, ART is mostly or at least partly a private service paid for out of pocket. A large share of the population struggles to fund and access it [33–36]. These research directions could be particularly interesting in the USA and China and in less wealthy nations that offer only modest or no public funding of ART. These issues affect the great majority and have immediate implications for decision-makers in every country. We therefore argue that this gap in the literature dealing with access to treatment, as well as the burden of ART funding on households, is problematic and should be addressed by redirecting research attention to these challenges.

Overall, our analysis revealed that the ELSI of ART literature exhibits a preference for areas of study that represent conceptual challenges and attract philosophical analysis, often involving thought experiments and discussions of hard or extreme cases. There is also a predilection for areas that permit access to relatively straightforward empirical research. However, the literature seems less inclined to address systemic justice issues that necessitate economic analysis and engagement with challenges of barriers to access.

We believe that while conceptual analysis and thought experiments hold value and are interesting, an excessive focus on these aspects can be problematic. This is particularly concerning when it consumes most of the intellectual and research resources in the field, leaving less room for studies that explore practical issues such as cost and associated analyses of justice and equity of access, or studies addressing knowledge and attitudes, and their potential impact on infertility prevention or the use and misuse of ART. We argue that a more balanced approach to ELSI of ART research is needed, incorporating both philosophical discussions and the exploration of practical concerns that directly affect the lives of those dealing with infertility and ART.

### Study limitations and future research directions

Our analysis is not exempt from some methodological limitations. First, our selection and cleaning process necessarily contains a certain level of subjectivity. The selection criteria were complex and included terms selected by the authors. Many articles were removed by “manually” analyzing their abstracts and both false-positives and false-negatives are possible. In particular, we made an effort to avoid amassing a large number of medical-technical articles (false-positive) by excluding certain terms (both MeSH-terms and keywords) from our queries. The exclusion might have led to fewer articles appearing under the “Cost and Outcome” topic (false-negative), which could explain the low share of articles associated with this topic. Second, in the classification process, the ART fields were defined according to keywords’ selection, while the occurrence of one term in the abstract indicated an affiliation to an ART field. This could lead to some mistakes. Third, a country was defined as the research subject based on its being mentioned in the abstract (including names of cities). We made a great effort to manually identify and correct mistakes resulting from this methodology. However, we assume that few errors could have remained unnoticed.

Finally, topic modeling by LDA has some limitations [37]. The algorithm assumes a certain probabilistic distribution behind the word/article association, which may not hold in reality. Moreover, there is no “standard” or objective way of fixing the number of topics, which remains a free parameter, adjusted in accordance with different metrics. Consequently, due to the number of topics one eventually selects, some smaller topics may remain hidden. Despite these limitations, our findings are based on a comprehensive corpus collected from three major databases, a relatively strong LDA, and an additional method of categorization.

In terms of future research, we may further exploit the collected database by dividing it into ART fields and topics which enable various systematic literature reviews and meta-analyses by extracting a single ART field (or topic) and presenting its distributions of study designs, key issues, research questions, and outcomes, according to geographic location and timeline. Furthermore, building upon our findings, future research could investigate the factors contributing to the shifts in research focus across various fields and topics that we have demonstrated, as well as explore some of the hypotheses we have raised. By examining the underlying reasons for these shifts, their relationships with ELSI research trends, and potential policy implications, scholars can deepen the understanding of the dynamics within the broader context of assisted reproduction. Finally, future studies can explore whether local issues (that emerge from local policies or socio-economic contexts) are sometimes consequently studied in other countries or regions.

## Conclusion

This paper analyzed the body of academic literature exploring international research on ELSI of ART emphasizing the importance of diversifying cultural perspectives and enriching the global discussion on the topic. Our findings demonstrate that international research can offer valuable insights and identify gaps and opportunities that would otherwise remain unnoticed. We noted a geographic centralization in terms of research production, reflecting an unequal distribution of research fund across countries and regions. Given the potential of international research to challenge local social values, ask novel questions, and foster international collaborations, we conclude that it is important to encourage researchers from wealthy academic centers to collaborate with researchers from regions with fewer resources, and to focus on less explored regions.

Some of the largest research gaps we identified concern China and Japan, the two world leaders in ART cycles, and Russia, another major ART user. Their unique cultural contexts yield particular restrictions on some types of ART, which in turn produce unique conditions for cross-border reproductive care. This highlights the importance of international research in examining the impact of cultural differences on ART practices and regulations. While the observed gap can be partially explained based on language and place of publication (we only considered publications with abstracts in English and there may be substantial scholarship on the ELSI of ART published in local journals), it remains important to increase the research attention given to these countries and their cultures, considering their leadership in clinical use.

We advocate for a more balanced approach in ELSI of ART research, incorporating both philosophical discussions and the exploration of practical concerns that directly affect the lives of patients, couples, and individuals requiring ART services. International research plays a crucial role in addressing the diverse needs and challenges faced by different populations, particularly in the context of cost, access, and the economic burden created by ART. Yet, these topics remain underexplored compared with topics that only touch on a portion of ART cycles (such as sperm and egg donation, surrogacy, or PGT for sex selection) and to which the literature dedicates much attention. We therefore offer a call to the ELSI of ART research community to divert more attention to questions of cost and access, while emphasizing the value of international research in uncovering unique issues and fostering diverse perspectives.

The same is true for issues of knowledge about and attitudes towards infertility and ART, which are exceptionally important yet understudied. International research in this area can provide valuable insights into the global variations in attitudes and knowledge. Future research could explore the level of interest this body of literature has in providing policy recommendations and guidance, across regions and topics. Much of the empirical and the conceptual research on ELSI of ART has strong normative implications. It would thus be interesting to explore whether researchers take the extra step and turn these implications into normative recommendations that can inform and improve policy, in order to provide better protections to all stakeholders in the ART space, but especially to vulnerable parties.

## Appendix A. Inclusion and exclusion criteria for articles in the review

**Table:**

**Inclusion criteria**	**Exclusion criteria**
All articles concerning all sorts of ART and Assisted Insemination (AI) which are dealing either: • Fully or partially with demographic research (national or regional activity reports and registries, geographical and socio-economic patterns of usage), ELSI, economic, regulations, anthropological, and political issues. OR • with value-based approaches, attitudes, opinions, expectations, and preferences. OR • with education, knowledge, and empowerment of decision-making among patients and donors. OR • with psychology and quality of life issues, raising ELSI or affecting government policy making and patients’ decision-making.	Articles which focus on either: • Clinical methods/outcome or technical issues with no relation to ELSI or social sciences. OR • Physicians’ professional approaches, opinions, preferences, education, and knowledge concerning clinical decision-making of technical character. OR • Psychology/quality of life outcomes by way of empirical research with no ELSI or impact on patients’ decision-making.
Rejection criteria: Articles dealing exclusively with (1) Animal research; (2) Treatment outcome, unless measured in terms of population and socio-economic characteristics (i.e., national registries); (3) Clinical policies on a local level (in contrast to national/regional policies); (4) Clinical outcome and performance of technologies and protocols; (5) Processes within hospitals and clinics; (6) Prenatal testing and selection; (7) Therapeutics (non-ART) uses of stem cell research; (8) Clinical trials or reports in psychiatry/psychology with no ELSI or impact on patients’ decision-making.	

## Appendix B. MeSH-terms and keywords

**Table:**

**Group A — Inclusion of ART terms**	**MeSH-terms (for PubMed) (28)**
	Reproductive Techniques, Assisted • Donor Conception • Embryo Transfer • Single Embryo Transfer • Fertility Preservation • Fertilization in Vitro • Mitochondrial Replacement Therapy • Sperm Injections, Intracytoplasmic • Gamete Intrafallopian Transfer • Insemination, Artificial • Insemination, Artificial, Heterologous • Insemination, Artificial, Homologous • Oocyte Donation • Oocyte Retrieval • Posthumous Conception • Embryo Disposition • Sperm Banks • Surrogate Mothers • Preimplantation Diagnosis • Sex Preselection • Gene Editing • Genome Editing • Genetic Enhancement • Adult Germline Stem Cells • Germ Cells • Ovum • Oocytes • Embryo Research • Research Embryo Creation
	**Keywords (for WoS and Scopus) (38)**
	assisted reprod* • assisted procreat* • reproductive techn* • in vitro fertili* • intracytoplasmic sperm injection • In vitro gametogenesis • egg don* • oocyte don* • sperm don* • embryo don* • donor eggs • donor conception • fertility preservation • oocyte cryopreserv* • egg freez* • sperm cryopreserv* • embryo cryopreserv* • oncofertility • leftover embryos • surplus embryos • frozen embryos • preimplantation genetic • reproductive genetic* • germline engineering • germline gene editing • germline gene modification • germline genetic modification • mitochondrial replacement • mitochondrial don* • surrogacy • surrogate mother • gestational carrier • uterus transplantation • artificial insemination • donor insemination • posthumous insemination • posthumous conception • posthumous reproduct*
**Group B — Inclusion of terms from disciplines of humanities and social sciences**	**MeSH-terms (for PubMed) (23)**
	Disability Evaluation • Anthropology • Demography • Economics • Forecasting • Policy • Private Sector • Public Sector • Sociology • Work-Life Balance • Education • Stakeholder Participation • History • Knowledge • Philosophy • Religion • Disabled Persons • Vulnerable Populations • Population Characteristics • Health Care Economics and Organizations • Health Services Administration • Health Care Quality, Access, and Evaluation • Attitude
	**Keywords (for WoS and Scopus) (28)**
	*ethic* • *access* • anonym* • attitude* • perception • *consent* • market* • crossborder • disclos* • eugen* • *identity • justi* • educat* • law* • legis* • legal* • moral* • inmoral • policy* • politic* • govern* • sex select* • touris* • view* • autonom* • desire* • vulnerab* • relatedness
**Group C — Exclusion of medical-technical terms**	**MeSH-terms (for PubMed) (45)**
	Ovulation Induction • Neoplasms • Pregnancy Complications • Follicle Stimulating Hormone • Breast Neoplasms • Odds Ratio • Sperm Motility • Prognosis • Semen Analysis • Congenital Abnormalities • Gonadotropin-Releasing Hormone • Ovarian Hyperstimulation Syndrome • Polycystic Ovary Syndrome • Pedigree • Recombinant Proteins • Clomiphene • Zygote • Mice • Reproducibility of Results • Ovarian Follicle • Chorionic Gonadotropin • Sperm Retrieval • Ovarian Neoplasms • Testis • Gonadotropins • Estradiol • Randomized Controlled Trials as Topic • Oligospermia • In Vitro Oocyte Maturation Techniques • Superovulation • Zygote Intrafallopian Transfer • Polar Bodies • Zona Pellucida • Sperm Head • Acrosome • Sperm Tail • Spermatids • Spermatocytes • Spermatogonia • Embryonic Germ Cells • Plants • Case-Control Studies • Botany • Agriculture • Clinical Trials as Topic
	**Keywords (for WoS and Scopus) (38)**
	ovulation Induction • neoplasms • follicle stimulating hormone • odds ratio • sperm motility • semen analysis • congenital abnormalities • gonadotropin • ovarian hyperstimulation • polycystic ovary • pedigree • recombinant proteins • clomiphene • zygote • ovarian follicle • testis • estradiol • randomized controlled trials • controlled trials • clinical trials • controlled clinical trial • validation study • polar bodies • zona pellucida • sperm head • acrosome • sperm tail • spermatids • spermatocytes • spermatogonia • embryonic germ cells • plants • case control • botany • agriculture • oligospermia • oocyte maturation • superovulation

*Zero or more characters

## Appendix C. Words and terms for division into ART fields

**Table:**

Axis A	
Egg Donation	biobanking of eggs and embryo • buying and selling gametes embryos • child donor • donated egg • donated gamete • donated oocyte • donating eggs • donor art • donor child • donor conceived offspring • donor conception • donor egg • donor gamet • donor of gamet • donor of sperm or egg • donor offspring • donor oocyte • donor registry • donor sibling • egg bank • egg don • egg shar • eggdonation • gamete bank • gamete don • gametes don • gametes sale • half sibling • oocyte bank • oocyte don • oocytes don
Sperm Donation	buying and selling gametes embryos • child donor • donate his sperm • donated gamete • donated sperm • donating sperm • donation of gamete • donor art • donor child • donor conceived offspring • donor conception • donor father • donor gamet • donor insemination • donor of gamet • donor of sperm or egg • donor offspring • donor semen • donor sibling • donorconceived child • gamete bank gamete don • gametes don • gametes sale • half siblings • known donors • semen bank • semen donor • single mother • sperm bank • sperm don • sperm from a known donor • spermdon
Embryo Donation	14 day limit • s biobanking of eggs and embryo • s buying and selling gametes embryos • s donated eggs and embryo • s donated embryo • s donation embryo embryo adoption • s embryo don • s embryodon • s embryos in excess • s excess embryo • s frozen embryo • s surplus embryo
Surrogacy	commissioning parent • intended parent • shared gestation • subrog • surrog
Fertility Preservation	fp_ • cryopreserv • egg freez • embryo disposition • embryo freez • embryo storage • fertility preserv • fertilitypreserv • freezing egg • freezing oocyte • freezing ovari • frozen egg • frozen embryo • frozen sperm • not implanted embryo • oncofertility • oocyte preserv • ovarian tissue • post mortem • posthumous • postmortem • social freezing
Stem Cells	artificialgametes • embryo destructive research • embryo research • embryo scientific research • experimentation on embryos • gamete product • human embryo • oocytes for research • pluripotent • research on embryo • stem cell • totipot
Preimplantation Genetic Testing	_pgd_ • _pgs_ • _pgt_ • embryo select • enhancement • gender select • genetic counsel • genetic select • genetically select • hla matched • hla typing • pre implantation diagnos • pre implantatory exam • preimplantation diag • preimplantation or prenatal screening • preimplantational • preimplantatory • procreative benef • reproductive genetic • saviour sibling • selection of embryo • sex select • whole genome sequencing
Genome Editing and Cloning	_ggm_ • chimer • clone • cloning • crispr • designer baby • enhancement • gametogene • gene edit • genetic eng • genetic manipul • genetic modific • geneticeng • germ line • germline • nuclear transfer • posthuman • procreative benef • synthetic gamet
Mitochondrial Replacement Techniques	_mrt_ • mitochondrial disorder • mitochondrial gene trans • mitochondrial transfer • ooplasmic transfer • spindle transfer • three parent • tri parent
Assisted Insemination	_iui_ • insemination

*2267 articles were not assigned to any ART field and were labeled General (Gen)

## Appendix D. Spearman’s correlations between ART fields and topics

**Table:**

	Correlation coefficient
Egg Donation (S1) – Sperm Donation (S2)	0.521
Egg Donation (S1) – Family and Sociology (T5)	0.202
Sperm Donation (S2) – Assisted Insemination (S10)	0.337
Sperm Donation (S2) - Family and Sociology (T5)	0.341
Embryo Donation (S3) – Fertility Preservation (S5)	0.238
Surrogacy (S4) – Law and Religion(T4)	0.264
Fertility Preservation (S5) – Cost and Outcome (T2)	0.264
Fertility Preservation (S5) – Attitudes and Knowledge (T6)	0.264
Stem Cells (S6) – Genome Editing (S8)	0.233
Stem Cells (S6) – Bioethics (T1)	0.343
PGT (S7) – Genome Editing (S8)	0.213
PGT (S7) – Bioethics (T1)	0.265
Genome Editing (S8) – Bioethics (T1)	0.413
Assisted Insemination (S10) – Family and Sociology (T5)	0.216

*For all pairs, *p*-value (2-tailed) =0.000

## Appendix E. Topic modeling output

Topic 1 — Bioethics (green): (‘0.027*“ethical” + 0.015*“genetic” + 0.014*“moral” + 0.008*“medical” + “0.008*“legal” + 0.008*“ethic” + 0.007*“scientific” + 0.005*“principle” + “0.005*“development” + 0.005*“medicine”’).

Topic 2 — Cost and Outcome (bottom red): (‘0.040*“patient” + 0.019*“transfer” + 0.018*“rate” + 0.013*“cycle” + “0.009*“clinical” + 0.009*“procedure” + 0.008*“risk” + 0.008*“clinic” + “0.008*“cost” + 0.008*“ethical”’).

Topic 3 — Law and Policy (blue): (‘0.024*“law” + 0.019*“legal” + 0.011*“country” + 0.010*“social” + “0.009*“policy” + 0.008*“regulation” + 0.006*“legislation” + 0.006*“medical” “+ 0.006*“work” + 0.006*“analysis”’).

Topic 4 — Psychology (yellow): (‘0.029*“couple” + 0.015*“psychological” + 0.015*“infertile” + “0.010*“counselling” + 0.010*“factor” + 0.009*“patient” + “0.009*“participant” + 0.008*“social” + 0.008*“relationship” + “0.008*“interview”’).

Topic 5 — Family and Sociology (purple): (‘0.092*“child” + 0.049*“parent” + 0.041*“family” + 0.022*“couple” + “0.018*“mother” + 0.013*“lesbian” + 0.013*“conceive” + 0.012*“genetic” + “0.012*“sex” + 0.011*“relationship”’).

Topic 6 — Attitudes and Knowledge (top red) (‘0.028*“patient” + 0.020*“age” + 0.013*“couple” + 0.013*“risk” + “0.013*“attitude” + 0.012*“knowledge” + 0.009*“medical” + 0.008*“genetic” + “0.007*“young” + 0.007*“participant”’).

## Footnotes

1 Please note that a fully detailed description of the methods used in this study can be found in “Mapping Ethical, Legal, & Social Implications (ELSI) of Assisted Reproductive Technologies” (under review in *JARG*).

2 To low-rise and tokenize we used: https://tedboy.github.io/nlps/generated/generated/gensim.utils.simple_preprocess.html

3 “gensim.corpora.Dictionary”

4 I.e., transforming words/sentences into vectors (with 1 and 0): word: id mapping to apply TM afterwards. GENSIM topic modelling for humans. https://radimrehurek.com/gensim/corpora/dictionary.html

5 https://radimrehurek.com/gensim/models/coherencemodel.html for TM using gensim.models.LdaMulticore with the following parameters: gensim.models.LdaMulticore(corpus=corpus, id2word=id2word, num_topics=k, random_state=100, chunksize=100, passes=10, per_word_topics=True), k number of topics ranging from 2 to 15.

6 Information about author affiliation (with country of institution) was retrieved from the databases.

7 For example, “Portug” to capture Portugal and Portuguese, “Spain” and “Spani,” “Poland” and “Polish.” Also, cities’ names with double meaning (i.e., Male, Huntington) were carefully removed.

8 According to the United Nations Geoscheme: https://unstats.un.org/unsd/methodology/m49/ (some regions were merged due to proximity and low number of publications, i.e., Latin Americas and the Caribbean, Western and Southern Asia, Eastern Europe and Central Asia, Eastern and South-eastern Asia).

9 Minimum articles per country = 21, min cluster size =7 (Argentina, Iran and Malaysia were removed)

## Declarations

This research did not involve human participants or animals and did not require any informed consent of any type.
